# Continued smoking and posterior vitreous adhesion in the elderly evaluated on swept-source optical coherence tomography

**DOI:** 10.1038/s41598-020-75590-9

**Published:** 2020-10-28

**Authors:** Taku Toyama, Yohei Hashimoto, Hisashi Kawai, Kunihiro Azuma, Tomoyasu Shiraya, Fumiyuki Araki, Koichiro Sugimoto, Yutaka Watanabe, Hirohiko Hirano, Yoshinori Fujiwara, Kazushige Ihara, Hunkyung Kim, Satoshi Kato, Jiro Numaga, Shuichi Obuchi, Takashi Ueta

**Affiliations:** 1 Department of Ophthalmology, Graduate School of Medicine and Faculty of Medicine, The University of Tokyo, Tokyo, Japan; 2 Tokyo Metropolitan Institute of Gerontology, Tokyo, Japan; 3 Gerodontology, Department of Oral Health Science, Faculty of Dental Medicine, Hokkaido University, Sapporo, Japan; 4 Department of Social Medicine, Hirosaki University School of Medicine, Hirosaki, Japan; 5 Department of Ophthalmology, National Center for Global Health and Medicine, 1-21-1, Toyama, Shinjuku, Tokyo 162-8655 Japan

**Keywords:** Visual system, Vitreous detachment, Retinal diseases

## Abstract

In this cross-sectional study including 1150 eyes of 681 volunteers ≧ 65 years old without retinal pathology, factors affecting the progression of posterior vitreous detachment (PVD) were investigated. PVD stages were diagnosed based on swept-source optical coherence tomography (SS-OCT). Linear mixed effect model was used to determine whether age, gender, diabetes mellitus (DM), hypertension (HT), dyslipidemia (DL), and smoking status were associated with various stages of PVD. As a result, the multivariable analysis disclosed that the associations between older age and higher PVD stages (estimate [95% CI], 0.031 [0.020 to 0.042]; *P* < 0.0001), and current smokers and lower PVD stages (estimate [95% CI], − 0.24 [− 0.43 to − 0.056]; *P* = 0.011) were statistically significant. In contrast, female gender was not an independent factor affecting PVD stages in the elderly. Our analysis indicated that higher PVD stages observed in female eyes may be due to confounding effect, in which current smokers were predominantly males (i.e., 12.6% among males vs. 3.9% among females, *P* < 0.0001). In conclusion, our findings suggest that continuous smoking is associated with an adherent vitreoretinal interface in the elderly.

## Introduction

The detachment and adhesion of the posterior vitreous are involved in various vitreoretinal disorders. Posterior vitreous detachment (PVD) can often lead to the development of rhegmatogenous retinal detachment^1^, macular hole^2^, and epiretinal membrane (ERM)^3^, whereas persistent vitreoretinal adhesion contributes to the pathology of vitreomacular traction syndrome^4^, diabetic retinopathy (DR)^5^, age-related macular degeneration (AMD)^6^, and uveitis^7^. PVD has traditionally been observed through slit-lamp ophthalmoscopy and ultrasonography, However, recently, more detailed examination on the vitreoretinal interface has been observed through swept-source optical coherence tomography (SS-OCT) that visualize the structure of vitreous cortex in detail^8–11^.

Previous studies have identified the predisposing factors for the development of PVD. Older age is the most important factor associated with PVD^12,13^. Female gender has also been reportedly attributed to PVD^12,13^. PVD starts to progress in some individuals in their 40 s, and the prevalence suddenly increases in their 60 s^10,13^. High myopia is associated with PVD, especially at a younger age, while the association at an older age might not be certain^14^. Menopause and higher intake of vitamin B6^15^ have also been suggested to be contributing factors of PVD.

Smoking contributes to the pathogenesis of ocular diseases involving the posterior segment of the eye, including AMD, polypoidal choroidal vasculopathy, macular edema, uveitis, and scleritis, which may be due to its pro-inflammatory activities^16^. The possible effect of smoking on the development of PVD has not been fully understood to date^14,15^, and there is especially a lack of detailed evaluation of its effects on the vitreoretinal interface using SS-OCT.

In this present study, a community-dwelling elderly population in Tokyo was enrolled, and potentially predisposing factors for the development of PVD in the elderly ≧ 65 years old were investigated through SS-OCT.

## Methods

This cross-sectional study was conducted in 2018 as part of a prospective cohort study that has started in 2011 enrolling the community-dwelling elderly population in the Itabashi ward, a northwestern district of Tokyo. The study was approved by the institutional review board and ethics committee of the Tokyo Metropolitan Institute of Gerontology^17–19^, and conducted according to the declaration of Helsinki. Written informed consent was provided by the participants. Some parts of the methods in the present study is similar to those of our recent study^20^, as described below.

### Participants

In this study, volunteers aged 65 years or older underwent annual comprehensive clinical examinations, including blood tests, body composition measurements, cognitive function tests, as well as comorbidity, and social background questionnaires. In 2018, in addition to these annual comprehensive examinations, the participants underwent fundus color photography, SS-OCT, and SS-OCT angiography (SS-OCTA) conducted by experienced optometrists who were not aware of the purpose of this present study.

For the current study, the exclusion criteria were as follows: (1) eyes with a history of retinal disease (i.e., DR, retinal vein occlusion, AMD, glaucoma, myopic maculopathy, and epiretinal membrane), as reported by the participants through a questionnaire or demonstrated by fundus photos, SS-OCT, or SS-OCTA obtained through the present study; (2) OCT images with insufficient quality, as measured by a built-in software (i.e., an image quality (IQ) metric of 40 or less)^21^.

### OCT image acquisition and PVD stage

**Figure 1:**
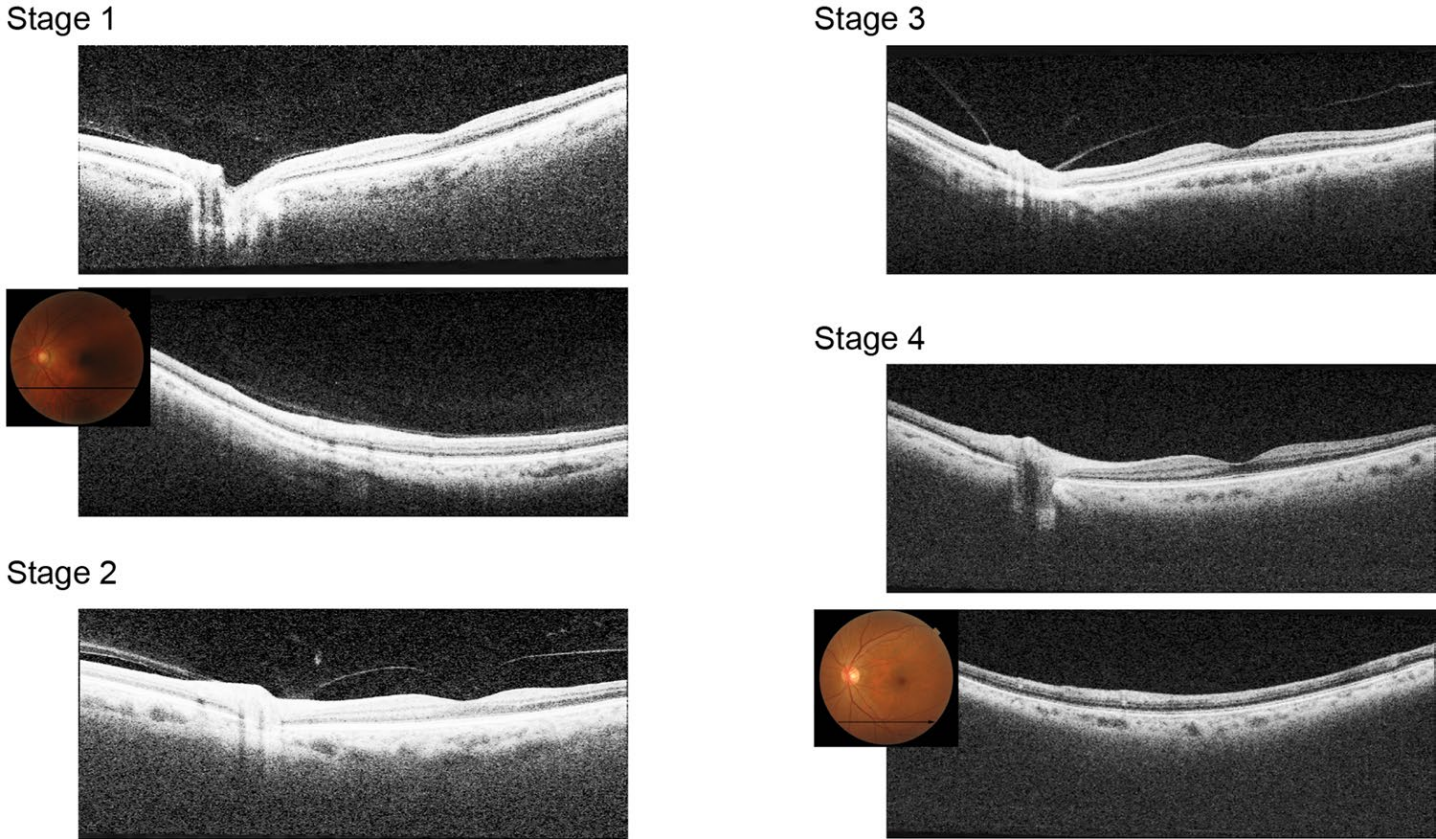
PVD stages observed on SS-OCT. In stage 1, adhered vitreous with premacular bursa and area of Martegiani (arrowhead) is observed. In the outer macular area, vitreous cortex can be visualized. In stage 2, PVD occurs except persistent adhesion to the papilla and fovea. In stage 3, PVD occurs except persistent adhesion to the papilla. In stage 4, premacular bursa, area of Martegiani, or vitreous cortex is not observed.

Two trained operators captured OCT images of the participants using SS-OCT (DRI OCT Triton; Topcon Inc., Tokyo, Japan) for each eye. It uses a tunable laser as a light source to provide a 1050-nm-centered wavelength. This device reaches a scanning speed of 100,000 A-scans per second, yielding 8 and 20 μm axial and transverse resolution in tissue, respectively. The SS-OCT images were obtained in two ways. The first was a 12 mm × 9 mm area scan with a density of 512 × 256 centered on the fovea, and covered the macula and optic nerve disc. The second is a single horizontal line scan through the fovea and optic nerve, in which a built-in signal averaging function worked more effectively to visualize structures in the vitreous. In addition, to enhance visualization of the vitreous structures, contrast was adjusted by the mode of enhanced vitreous visualization (EVV)^8,9^. The extent of PVD was graded based on a recently proposed system based on SS-OCT^10^: stage 0 = no PVD; stage 1 = PVD at mid-periphery and subtle vitreous separation in the posterior retina; stage 2 = PVD, except for persistent adhesion to the papilla and fovea; stage 3 = PVD, except for persistent adhesion to the papilla; and stage 4 = complete PVD in the posterior retina. Only two eyes were found to be possible stage 0 (i.e., no vitreous cortex separation and detecting premacular bursa). However, as SS-OCT images of peripheral fundus were not acquired, we did not differentiate between PVD stages 0 and 1 as shown in a recent study^11^. However, because stage 0 PVD in the eyes ≧ 60 years has been shown to be rare^10^, eyes were categorized into PVD stages of 1, 2, 3, or 4 (Fig. 1). Three experienced retina physicians (T.T., F.A., T.U.) who were masked to other information of the subjects determined the stages of PVD independently, and disagreement on the PVD staging was discussed and resolved by three physicians.

### Medical and smoking history

History of diabetes mellitus (DM) was defined as fasting blood glucose ≧ 126 mg/dL, HbA1c ≧ 6.5%, or being on medication for DM. History of hypertension (HT) was regarded as systolic/diastolic pressure of ≧ 140/90 mmHg or being on medication for HT. History of dyslipidemia (DL) was defined as serum low-density lipoprotein (LDL) cholesterol ≧ 140 mg/dL, serum high density lipoprotein (HDL) cholesterol < 40 mg/dL, serum triglyceride ≧ 150 mg/dL, or being on medication for DL. Smoking status was categorized as current, past, or non-smokers. Past smokers were defined as those who quitted smoking 1 year ago or earlier, and current smokers were defined as those who continued smoking or quitted within 1 year.

### Statistics

Statistical analysis was performed using the JMP Pro 14 software (SAS). To include both eyes of the same participant in the analysis, linear mixed model was used. The linear mixed model is equivalent to ordinary linear regression in that the model describes the relationship between the predictor variables and a single outcome variable. However, standard linear regression analysis makes the assumption that all observations are independent of each other. In the current study, measurements are nested within subjects and thus dependent of each other. Ignoring this grouping of the measurements will result in the underestimation of standard errors of regression coefficients. Therefore, the linear mixed model adjusts for the hierarchical structure of the data^22,23^.

The effects of age, gender, DM, HT, and DL, and smoking status (i.e., current, past, or non-smoker) were assessed using univariable and multivariable analysis to explore their possible effects on PVD stages. The summarized numerical data were presented as mean ± standard deviation (SD). The effect of each explaining variable was described as an estimate with 95% confidence interval (CI). A *P *value of < 0.05 was regarded as statistically significant.

## Results

In total, 1150 eyes of 681 participants were included for this study. Table 1 shows the characteristics of the eyes stratified by their PVD stage. Among the 1150 eyes included, 152 (13.2%), 49 (4.3%), 49 (4.3%), and 900 (78.3%) were categorized into PVD stages 1, 2, 3, and 4, respectively. Univariable analysis detected significant differences in the distributions of age, sex, and smoking status across the different PVD stages. Older age and female gender were found to be significantly associated with higher PVD stages, whereas eyes of current smokers were identified to frequently have lower PVD stages. In contrast, no significant difference was detected in the distributions of DM, HT, or DL. The OCT image quality (IQ) metric was equivalent across different PVD stages.

**Table 1:** Characteristics of participants stratified by PVD stage.

	Stage 1	Stage 2	Stage 3	Stage 4	*P* value
Eyes, n	152	49	49	900	
Age, mean ± SD	70.3 ± 0.5	69.7 ± 0.9	71.5 ± 0.9	73.7 ± 0.2	< 0.0001*
**Sex, n(%)**					
Male	79 (52.0%)	19 (38.8%)	16 (32.7%)	322 (35.8%)	0.0046*
Female	73 (48.0%)	30 (61.2%)	33 (67.3%)	578 (64.2%)	
DM, n(%)	17 (11.2%)	6 (12.2%)	9 (18.4%)	96 (10.7%)	0.85
HT, n(%)	68 (44.7%)	16 (32.7%)	19 (38.8%)	358 (39.8%)	0.77
DL, n(%)	61 (40.1%)	20 (40.8%)	14 (28.6%)	339 (37.7%)	0.85
**Smoking, n(%)**					
Current	23 (15.1%)	7 (14.3%)	1 (2.0%)	52 (5.8%)	0.0025*
Past	55 (36.2%)	16 (32.7%)	13 (26.5%)	242 (26.9%)	0.62
Never	74 (48.7%)	26 (53.1%)	35 (71.4%)	606 (67.3%)	NA
IQ, mean ± SD	61.2 ± 0.6	59.1 ± 1.0	60.0 ± 1.0	60.2 ± 0.2	0.21

*DM* diabetes mellitus, *HT* hypertension, *DL* dyslipidemia, *IQ* OCT image quality metric, *SD* standard deviation, *NA* not analyzed.

P* < 0.05.

To address the independent association between these variables and PVD stages, multivariable analysis was performed with the objective variable being the PVD stage and the explanatory variables being their age, sex, DM, HT, DL, and smoking status (Table 2). The analysis noted that older age was significantly and independently associated with higher PVD stages (estimate [95% CI], 0.031 [0.020 to 0.042]; *P* < 0.0001), which is consistent with the previous studies. Female gender was no longer a significant factor associated with PVD stages in multivariable analysis. DM, HT, or DL was not associated with PVD stages, which is also consistent with the previous reports. Notably, current smokers were identified to have significantly lower PVD stages compared to non-smokers (estimate [95% CI], − 0.24 [− 0.43 to − 0.056]; *P* = 0.011). In contrast, smoking in the past did not affect PVD stages compared to non-smokers.

**Table 2:** Univariable and multivariable evaluation of factors contributing to higher PVD stages.

	Univariable analysis		Multivariable analysis	
	Estimate (95%CI)	*P* value	Estimate (95%CI)	*P* value
Age	0.033 (0.022 to 0.044)	< 0.0001*	0.031 (0.020 to 0.042)	< 0.0001*
Female gender	0.11 (0.034 to 0.19)	0.0046*	0.046 (− 0.041 to 0.13)	0.30
DM	− 0.012 (− 0.13 to 0.10)	0.85	0.0010 (− 0.12 to 0.11)	0.99
HT	− 0.011 (− 0.087 to 0.064)	0.77	− 0.028 (− 0.10 to 0.049)	0.48
DL	− 0.0074 (− 0.084 to 0.069)	0.85	− 0.021 (− 0.097 to 0.055)	0.58
**Smoking**				
Current	− 0.29 (− 0.49 to − 0.10)	0.0025*	− 0.24 (− 0.43 to − 0.056)	0.011*
Past	0.034 (− 0.099 to 0.17)	0.62	0.066 (− 0.070 to 0.20)	0.34
Never	Ref		Ref	

*DM* diabetes mellitus, *HT* hypertension, *DL* dyslipidemia, *CI* confidence interval.

P* < 0.05.

Next, in order to rule out the effect of cataract surgery on PVD progression, we conducted the same multivariable analysis excluding 191 eyes with history of cataract surgery. The analysis showed consistent results in which older age was associated with higher PVD stages (estimate [95% CI], 0.031 [0.018 to 0.044]; *P* < 0.0001), and current smokers were associated with lower PVD stages (estimate [95% CI], − 0.33 [− 0.54 to − 0.12]; *P* = 0.0019), while no significant effect was seen in sex, DM, DL, or HL.

In terms of assessing the association between female gender and higher PVD stages, the univariable analyses revealed a significant association, while multivariable analyses did not, suggesting an influence of confounding effect. Indeed, a higher prevalence of current smokers in men than in women was found. The number of current smokers in women was 28 out of 714 (3.9%) while that in men was 55 out of 436 (12.6%) (*P* < 0.0001 by chi-square test). Therefore, multivariable analysis was performed using the objective variables of PVD stages relative to the explanatory variables in current smokers only (n = 83), past smokers only (n = 326), and non-smokers only (n = 741). After adjusting for age, DM, HT, and DL, the associations of female sex by estimate (95% CI; *P* value) among current, past, and non-smokers were determined to be − 0.0072 (− 0.39 to 0.38; *P* = 0.97), 0.032 (− 0.14 to 0.20; *P* = 0.72), and 0.071 (− 0.032 to 0.17; *P* = 0.18), respectively (Tables 3, 4, 5). The results suggest that sex might not be an independent factor affecting PVD stages in the elderly.

**Table 3:** Univariable and multivariable evaluation of factors contributing to higher PVD stages in current smokers (n = 83).

	Univariable analysis		Multivariable analysis	
	Estimate (95%CI)	*P* value	Estimate (95%CI)	*P* value
Age	0.097 (0.032 to 0.16)	0.0041*	0.099 (0.031 to 0.17)	0.0053*
Female gender	0.052 (− 0.35 to 0.46)	0.80	− 0.0072 (− 0.39 to 0.38)	0.97
DM	− 0.044 (− 0.62 to 0.53)	0.88	− 0.16 (− 0.73 to 0.40)	0.56
HT	0.23 (− 0.15 to 0.61)	0.23	0.012 (− 0.38 to 0.40)	0.95
DL	0.41 (0.0056 to 0.81)	0.047*	0.41 (0.016 to 0.80)	0.042*

*DM* diabetes mellitus, *HT* hypertension, *DL* dyslipidemia, *CI* confidence interval.

P* < 0.05.

**Table 4:** Univariable and multivariable evaluation of factors contributing to higher PVD stages in past smokers (n = 326).

	Univariable analysis		Multivariable analysis	
	Estimate (95%CI)	*P* value	Estimate (95%CI)	*P* value
Age	0.041 (0.019 to 0.063)	0.0004*	0.044 (0.022 to 0.66)	0.0001*
Female gender	− 0.017 (− 0.20 to 0.16)	0.85	0.032 (− 0.14 to 0.20)	0.72
DM	0.047 (− 0.16 to 0.26)	0.66	− 0.13 (− 0.078 to 0.34)	0.22
HT	− 0.084 (− 0.24 to 0.072)	0.29	− 0.070 (− 0.23 to 0.089)	0.39
DL	− 0.20 (− 0.36 to − 0.042)	0013*	− 0.23 (− 0.40 to 0.064)	0.007*

*DM* diabetes mellitus, *HT* hypertension, *DL* dyslipidemia, *CI* confidence interval.

P* < 0.05.

**Table 5:** Univariable and multivariable evaluation of factors contributing to higher PVD stages in never smokers (n = 741).

	Univariable analysis		Multivariable analysis	
	Estimate (95%CI)	*P* value	Estimate (95%CI)	*P* value
Age	0.020 (0.0075 to 0.032)	0.0017*	0.019 (0.066 to 0.032)	< 0.0030*
Female gender	0.084 (− 0.019 to 0.19)	0.11	0.071 (− 0.032 to 0.17)	0.18
DM	− 0.012 (− 0.15 to 0.13)	0.87	− 0.026 (− 0.17 to 0.12)	0.72
HT	0.019 (− 0.065 to 0.10)	0.65	0.00017 (− 0.085 to 0.086)	1.0
DL	0.029 (− 0.054 to 0.11)	0.49	0.020 (− 0.063 to 0.10)	0.64

*DM* diabetes mellitus, *HT* hypertension, *DL* dyslipidemia, *CI* confidence interval.

P* < 0.05.

The other possible explanation why we observed a significant association between female gender and higher PVD stages only in univariable analysis may be due to higher age in female subjects, and the mean ± SD age was 73.5 ± 6.5 years old in females and 72.2 ± 6.5 in males, *P* = 0.0010). Therefore, we performed univariable analysis excluding eyes of females aged 85 years or older. In this condition, the mean ± SD age of female subjects down to 72.8 ± 5.8 years old, while the analysis consistently showed that female gender was associated with higher PVD stages (estimate 0.10 [95%CI 0.027–0.18], p = 0.0084).

Lastly, interaction between smoking status and gender was tested (Table S1), which did not indicate a significant interaction between the two explanatory variables.

## Discussion

This present study has examined the factors associated with vitreous detachment or adhesion in the elderly using SS-OCT. We found that even in the elderly, higher age was associated with the development of PVD. In addition, we also observed a significant association between continued smoking and an adhered vitreous, while smoking in the past did not affect vitreous detachment or adhesion.

Our observation of lower PVD stages in current smokers suggests a possible role of continued smoking in inhibiting PVD. Smoking has inflammatory activity^24^. Although the mechanism of how smoking leads to inflammation remains inconclusive, recent studies suggest that nicotine can activate neutrophil extracellular trap (NET) formation, which can accelerate inflammation throughout the body^25,26^. Smoking is associated with ocular disorders in which inflammation plays an important pathogenic role, including AMD, uveitis, and scleritis^16^. In eyes with neovascular AMD, persistent vitreomacular adhesion was more frequently observed and considered concurrent with low-grade inflammation^27–29^. In eyes with non-infectious uveitis, PVD was associated with a more favorable treatment response, implying a pro-inflammatory role of vitreomacular adhesion^7^. Therefore, we speculate that continued smoking may cause sustained low-grade inflammation at the vitreoretinal interface, which suppresses separation of the vitreous form the retinal surface.

Several studies have examined the association between smoking status and PVD development. A previous case–control study enrolled 138 patients with acute PVD and 114 control patients without PVD^15^. The subjects were ≧ 35 years old with a mean of 62 years, and PVD was diagnosed using 90D biomicroscopy and B-scan ultrasonography. In the study, the authors suggested that smoking was not associated with PVD, however, providing no data conforming this claim. Compared to the present study, the enrolled participants were younger, diagnostic techniques for PVD were different, and a different statistical method (i.e., logistic regression with backward selection) was used. Another study was a population-based study in northern China^14^. It enrolled 5890 participants, and the age of the subjects was ≧ 30 years old, with a mean of 52 years. PVD was diagnosed using 90D biomicroscopy and B-scan ultrasonography. Smoking status was analyzed as current, past, or non-smoker. A multivariable logistic regression model did not reveal a significant association between smoking and PVD. Compared to the present study, there were differences in the study design and the subjects’ age. Moreover, there might have been a sensitivity issue to detect PVD, PVD had been diagnosed in only 8.4% of eyes in a population of ≧ 60 years old using biomicroscopy and ultrasonography. In the present study, stage 4 PVD was diagnosed in 78.3% of the enrolled eyes ≧ 65 years. Consistently, in a recent study, stage 4 PVD was diagnosed in 78.5% of the eyes aged ≧ 60 years using SS-OCT^13^. Similarly, a previous study reported 67.4% of the eyes ≧ 70 years old have late-stage PVD using ultrasonography and spectral-domain OCT^30^. The other study mentioning on smoking was a Beijing Eye Study^31^ in which the authors used spectral domain-OCT to find eyes with incomplete PVD. The limitation of SD-OCT with 6 mm scan is a difficulty to diagnose stage 4 PVD^32^, which might have affected the results especially when younger subjects were included in the study. Although how smoking was evaluated or categorized was not explained, smoking was reported to be significantly associated with incomplete PVD in univariable analysis, but not in multivariable analysis. Therefore, based on literature, the effects of smoking on PVD have remained unclear to date, and our results indicate a possible association of continued smoking in suppressing PVD progression in the elderly.

Intriguingly, a recent meta-analysis of multiple population-based studies revealed a significantly lower prevalence of ERM in current smokers compared to non-smokers, while no significant difference was found between past and non-smokers^33^. The mechanisms of lower ERM prevalence among current smokers remains unclear. Since ERM develops mostly in eyes with PVD, we speculate that continued smoking may prevent ERM formation possibly through its inhibitory effects on PVD, although a larger sample size is necessary to confirm the hypothesis.

The other issue raised by the present study is the role of gender for PVD progression. In literature, several studies using biomicroscopy and ultrasonography have reported an association between female gender and higher PVD prevalence^12,15,31^, while another study has reported no difference in PVD prevalence between men and women^30^. A recent study using SS-OCT has confirmed higher PVD stages in female eyes, which is consistent with our univariable analysis results^13^. Our results based on multivariable analysis unexpectedly suggested a confounding effect of smoking status in which current smokers are dominantly male. To eliminate the confounding effect by smoking status, analyses were performed in current, past, and non-smokers, separately. In each subgroup, there was no significant association between gender and PVD stages. The conflicting results among different studies may be due to different age groups of the subjects. In two studies (i.e. this study and a previous one^30^) in which no significant effect of gender on PVD was observed, the participants were older than those enrolled in the other studies^12,15,31^. In addition, a recent study compared the area size of vitreomacular attachment between male and female, and disclosed that a significantly smaller area size was observed in females. The study also showed that the gender effect became smaller in older age groups^34^. It suggests that PVD may progress earlier in females than in males, and PVD may be similarly at the final stage in both genders when evaluated in the age of ≧ 65 years old.

There have been various reports on the relationship between DM and PVD. In a population-based study, DM was determined not to be a predisposing factor for PVD^14^. On the other hand, another study reported that DM increases the area of vitreoretinal adhesions^35^. In response, a recent study reported that the presence or absence of DR, but not existence of DM alone, was the determining factor for PVD progression^5^. This present study, in which eyes with DR were excluded, indicates that DM without DR is unlikely to have an effect on PVD.

Several limitations exist in the present study. First, detailed ophthalmic examinations were not performed. Ophthalmic data were collected through questionnaires, fundus photographs, SS-OCT, and SS-OCTA. Images were carefully examined to exclude eyes with pre-existing retinal pathologies and characteristics of high myopia. Although high myopia is related to PVD in younger age groups, it might not be significant in those of older age^14^. Second, we included subjects aged ≧ 65 years, and the findings here are not applicable to younger populations. PVD was found to generally start to progress after the 40 s^36^, while prevalence of PVD drastically increases in the 60 s^10,13,14^. Therefore, the findings in this study are considered as a consequence of continued smoking for years. Third, the ethnicity of the subjects in this study was Japanese, and the findings here need to be tested in other ethnicities especially with different smoking habits. Forth, the number of eyes with PVD stages 2 or 3 were relatively low, which implies that the results are mainly based on the extremes of the PVD stages.

In summary, we evaluated factors associated with PVD stages in 1150 elderly eyes. Besides age, lower PVD stages were associated with current smokers. Our multivariable analyses indicate that gender itself is not an independent factor associated with PVD stages in the elderly. Instead, eyes of older females might only appear to be associated with higher PVD stages due to the lower prevalence of current smokers among women compared to men.

## Supplementary information

Supplementary Information

## Data Availability

All data generated or analyzed during this study are included in this published article and its Supplementary Information files.
