# Supplementary material for: Continued smoking and posterior vitreous adhesion in the elderly evaluated on swept-source optical coherence tomography
Source: Sci Rep. 2020 Oct 28;10:18460. doi: 10.1038/s41598-020-75590-9 (PMC7595182; doi:10.1038/s41598-020-75590-9)
Supplement: Supplementary file 1 — Supplementary Information [file 41598_2020_75590_MOESM1_ESM.pdf]

Continued smoking and posterior vitreous adhesion in the elderly evaluated on swept-source optical coherence tomography

Taku Toyama[1,2], Yohei Hashimoto[1], Hisashi Kawai[2], Kunihiro Azuma[1], Tomoyasu Shiraya[1], Fumiyuki Araki[1], Koichiro Sugimoto[1], Yutaka Watanabe[3], Hirohiko Hirano[2], Yoshinori Fujiwara[2], Kazushige Ihara[4], Hunkyung Kim[2], Satoshi Kato[1], Jiro Numaga[2], Shuichi Obuchi[2], Takashi Ueta[1,5]

[1]Department of Ophthalmology, Graduate School of Medicine and Faculty of Medicine, The University of Tokyo

[2]Tokyo Metropolitan Institute of Gerontology

[3]Gerodontology, Department of Oral Health Science, Faculty of Dental Medicine, Hokkaido University

[4]Department of Social Medicine, Hirosaki University School of Medicine

[5]Department of Ophthalmology, National Center for Global Health and Medicine

Table S1. Potentially influencing factors for higher PVD stages including gender-smoking interaction.

	Univariable analysis		Multivariable analysis	
	Estimate (95%CI)	P value	Estimate (95%CI)	*P* value
Age	0.033 (0.022 – 0.044)	<0.0001*	0.031 (0.020 – 0.042)	<0.0001*
Female gender	0.11 (0.034 – 0.19)	0.0046*	0.039 (-0.076 – 0.15)	0.51
DM	-0.012 (-0.13 – 0.10)	0.85	0.00064 (-0.12 – 0.12)	0.99
HT	-0.011 (-0.087 – 0.064)	0.77	-0.027 (-0.10 – 0.050)	0.49
DL	-0.0074 (-0.084 – 0.069)	0.85	-0.022 (-0098 – 0.054)	0.57
Smoking				
current vs. never	-0.29 (-0.49 – -0.10)	0.0025*	-0.24 (-0.44 – -0.034)	0.022*
past vs. never	0.034 (-0.099 – 0.17)	0.62	0.058 (-0.088 – 0.20)	0.44
Gender×smoking interaction				
gender×current (vs. never)	NA	NA	0.0015 (-020 – 0.20)	0.99
gender×past (vs. never)	NA	NA	-0.031 (-0.18 – 0.11)	0.67

DM; diabetes mellitus. HT; hypertension. DL; dyslipidemia. CI=confidence interval. NA=not analyzed. *P**<0.05.
